# Dependence of Graphene Oxide (GO) Toxicity on Oxidation Level, Elemental Composition, and Size

**DOI:** 10.3390/ijms221910578

**Published:** 2021-09-30

**Authors:** Tao Jiang, Carlo Alberto Amadei, Yishan Lin, Na Gou, Sheikh Mokhlesur Rahman, Jiaqi Lan, Chad D. Vecitis, April Z. Gu

**Affiliations:** 1 Department of Civil and Environmental Engineering, Northeastern University, Boston, MA 02115, USA; taojiangtony@gmail.com (T.J.); gouna0726@gmail.com (N.G.); mokhles@gmail.com (S.M.R.); gracelanjiaqi@gmail.com (J.L.); 2 John A. Paulson School of Engineering and Applied Sciences, Harvard University, Cambridge, MA 02138, USA; carloalberto.amadei@gmail.com (C.A.A.); vecitis@seas.harvard.edu (C.D.V.); 3 State Key Laboratory of Pollution Control and Resource Reuse, School of the Environment, Nanjing University, Nanjing 210046, China; 4 School of Civil and Environmental Engineering, Cornell University, Ithaca, NY 14853, USA; 5 Department of Civil Engineering, Bangladesh University of Engineering and Technology, Dhaka 1000, Bangladesh; 6 Institute of Materia Medica, Chinese Academy of Medical Sciences and Peking Union Medical College, Beijing 100050, China

**Keywords:** graphene oxide (GO), nanotoxicity, quantitative toxicogenomic assay, comet assay, reactive oxygen species (ROS) measurement assay

## Abstract

The mass production of graphene oxide (GO) unavoidably elevates the chance of human exposure, as well as the possibility of release into the environment with high stability, raising public concern as to its potential toxicological risks and the implications for humans and ecosystems. Therefore, a thorough assessment of GO toxicity, including its potential reliance on key physicochemical factors, which is lacking in the literature, is of high significance and importance. In this study, GO toxicity, and its dependence on oxidation level, elemental composition, and size, were comprehensively assessed. A newly established quantitative toxicogenomic-based toxicity testing approach, combined with conventional phenotypic bioassays, were employed. The toxicogenomic assay utilized a GFP-fused yeast reporter library covering key cellular toxicity pathways. The results reveal that, indeed, the elemental composition and size do exert impacts on GO toxicity, while the oxidation level exhibits no significant effects. The UV-treated GO, with significantly higher carbon-carbon groups and carboxyl groups, showed a higher toxicity level, especially in the protein and chemical stress categories. With the decrease in size, the toxicity level of the sonicated GOs tended to increase. It is proposed that the covering and subsequent internalization of GO sheets might be the main mode of action in yeast cells.

## 1. Introduction

Graphene-based materials (GNMs) possess outstanding electronic, thermal, and mechanical traits, making them one of the most promising engineered carbon-based nanomaterials (CNMs) [1,2,3]. Graphene oxide (GO) is one of the major GNMs that has been comprehensively exploited and investigated in recent years [4]. GO is a form of graphene with chemical modification and a high oxidation degree, which possesses colloidal stability in biologic media and carries a negative surface charge with the epoxide and hydroxyl functional group [5]. GO is applicable in various fields, such as hydrogen storage [6], catalysis [7], electrochemical devices [8], and separation membranes [9]. In addition, the biological applications of GO have been intensively studied recently because of their potential for bacterial inhibition [10], biosensors [11,12], and drug delivery vectors [13,14,15]. Although the application of GO would generate remarkable economic advantages, the mass production of GNMs unavoidably elevates the possibility of releasing them into the environment, raising public concern about their potential toxicological effects and the risks to humans and ecosystems. Moreover, these 2-D nanomaterials tend to dissolve in aquatic environments over time and are highly stable [16]. Thus, the assessment of GO toxicity is of great importance for environmental and human health.

The high surface reactivity exerted by GO [17], which facilitates biological and medical advancements, might also play an important role in their toxicological effects [18,19]. GO could potentially cause oxidative stress by generating reactive oxygen species (ROS) [20,21]. The oxidative stress is responsible for inducing lipid peroxidation and DNA damage, as well as the attachment of GO onto cell surfaces, thereby perturbing cell membranes and interacting with crucial metabolism-related biomolecules, resulting in toxicological impacts in the cells [5]. Previous studies also indicate immune response and toxicity in adult zebrafish induced by GO [22]. In addition, it is also reported that GO exposure can lead to cytotoxicity at high concentrations by inducing apoptosis and granuloma formation in human lungs [23].

It has been demonstrated that the physicochemical characteristics of GOs, such as size, purity, surface charge, functionalization, and the aggregation states, could also regulate its toxicological effects [24,25,26,27]. The effects of GO size on the distribution in mice in vivo was investigated [28], and the results suggest that small-sized and large-sized GOs accumulated mainly in different organs of the mice, indicating the size-dependent dispersion state of GO. Liu et al. studied the effect of the GO lateral size on toxicity in suspension using *E. coli* and concluded that the antimicrobial activity of GO decreased with the decreasing size in suspension [29]. However, the toxicity mechanisms might be largely distinct when applied to a different cell lines in vitro, especially eukaryotic cells that are more relevant to humans, where the relative size comparison might be different because of the larger dimensions of the eukaryotic cells.

The GO oxidation level can also exert an effect on its toxicity. The compatibility of GO with differing oxidation states was studied in vivo [30], suggesting that GO with a lower oxidation level leads to faster rates of infiltration, uptake, and clearance in immune cells after implantation. Guo et al. investigated the effects of GO and reduced GO (rGO) on biofilm formations and developments in Luria-Bertani (LB) media, employing *E. coli* and *S. aureus* as model microorganisms [31]. The results reveal that GO remarkably increased cell growth, as well as biofilm formations and developments under a concentration as high as 500 mg/L. In contrast, rGO (≥50 mg/L) significantly suppressed cell growth and biofilm formations. Nevertheless, only a few investigations have endeavored to elucidate the size and oxidation level effects, and the associated toxicity mechanisms are yet to be fully understood. A systematic evaluation of the impact of key GO physicochemical properties on its toxicity, particularly the molecular toxicity mechanisms, have not been reported.

One major challenge in nanotoxicity assessment is the daunting effort required to assess the huge, and ever-increasing number and diversity, of nanomaterials with great variances in their physiochemical properties and exposure matrices. Most nanotoxicity studies employ conventional toxicity assessment methods. Although these methods possess the benefits related to the anatomical similitudes of animals to humans, and their susceptibility to numerous health issues [32], utilizing animal tests in nanotoxicity evaluation is constrained by the large expense, intense workload, sophisticated analytical methodology, as well as long test lengths [33]. These drawbacks make the task for evaluating the tremendous number and diversity of nanomaterials nearly impossible. Accordingly, it is urgently needed for a systematic transition to include and tier the risk assessment system with higher throughput and mechanistic nanotoxicity testing methodologies [33,34,35].

Various nanotoxicity assessment studies have successfully applied omics-based techniques, such as genomics [36], proteomics [37,38], and metabonomics, that aim to globally and dynamically quantify the metabolic changes of living organisms in response to stimuli [39,40]. With the novel toxicogenomic-based high-throughput three-dimensional (exposure time, specific biomarker, and expression alteration magnitude) differential protein expression profiling approach employing a GFP-fused yeast reporter library, we have established and demonstrated its successful application for rapid, effective, and mechanistic toxicity evaluation [41,42,43,44]. Furthermore, the developed protein expression level index (PELI) allows for the generation of quantitative toxicogenomic endpoints that have been shown to correlate with conventional phenotypic endpoints [44,45,46,47,48,49].

In this study, we employed a quantitative toxicogenomic method to systematically evaluate the molecular toxicity profiles of GO and their dependence on the key physicochemical properties, including the oxidation level, elemental composition, and size. The toxicogenomic assay covers a wide range of biomarkers indicative of all main stress categories, including general, chemical, DNA, oxidative, and protein stress. Conventional phenotypic bioassays, including the reactive oxygen species (ROS) measurement assay for oxidative stress, and the alkaline comet assay for genotoxicity, were performed for toxicity endpoints comparison and phenotypic anchoring. The results reveal the toxicological effects and molecular toxicity mechanisms of GOs and disclose the potential relations between GO toxicity and its various physiochemical properties.

## 2. Results

### 2.1. Treatment and Characterization of Graphene Oxides

The physical and chemical characteristics of the five treated GO materials are shown in Table 1. The GOs were treated with strong acid and then purified to a great extent prior to use. No metal contaminations were observed for the GOs, and the impurities included 2–3% of sulfur and <1% of nitrogen. The oxygen contents for the untreated 45-min-sonicated, and 2.5-h-sonicated GOs were the same, which was 36.1 ± 0.1 %, while the UV-treated and thermally reduced GOs, with oxygen contents of 35 ± 0.7 % and 33.9 ± 0.1 %, respectively, had a lower oxygen than untreated GO. It is worth noting that the oxygen content of thermally reduced GO was significantly lower (*p* = 0.0001) than that of the untreated control GO, while the difference between the untreated control and the UV-treated GOs was not significant (*p* > 0.05). Correspondingly, the O/C ratio had the same trend for all five samples. The impurities and the monolayer content were the same for all five samples.

GO mainly consists of carbon and oxygen species bonded to carbon, e.g., the epoxy, hydroxyl, and carboxylic groups. Because of its complex nonstoichiometric nature, the contents for each species may vary significantly. In our study, untreated thermally reduced and sonicated samples have similar carbon-carbon, epoxy, hydroxyl, and carboxylic groups. In contrast, for the UV-treated sample, the carbon-carbon groups are significantly higher than for the untreated GO, while the epoxy and hydroxyl groups are significantly lower than for the untreated one.

**Figure 1:**
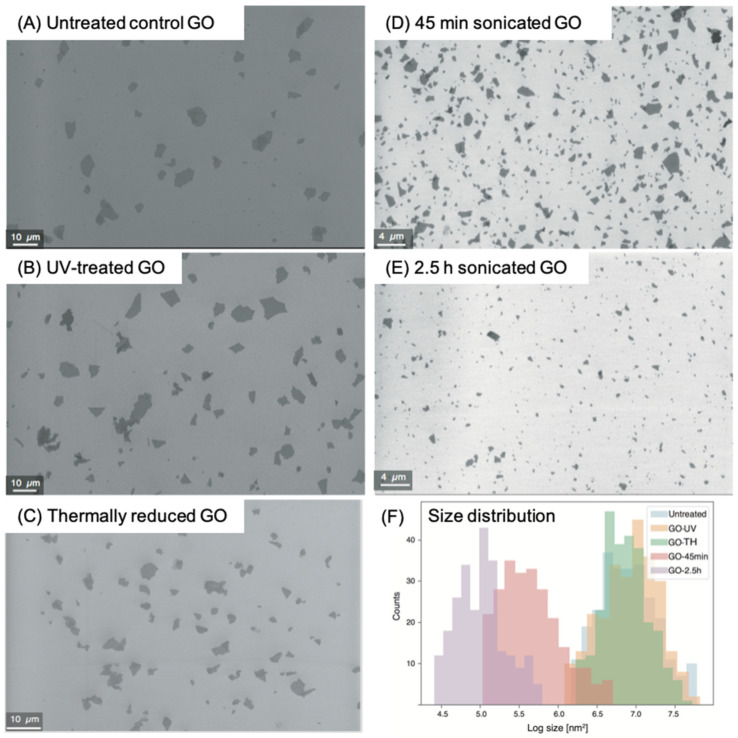
Graphene oxide (GO) morphology measured using a scanning electron microscope (SEM): (**A**) untreated control GO, Scale bar: 10 μm; (**B**) GO treated with 96 h UV, Scale bar: 10 μm; (**C**) GO treated with 96 h thermal reduction, Scale bar: 10 μm; (**D**) GO treated with 45 min bath sonication, Scale bar: 4 μm; (**E**) GO treated with 2.5 h bath sonication, Scale bar: 4 μm; and (**F**) size distribution of the 5 GO groups. The size distribution was obtained by image analysis software where >300 GO flakes were analyzed for each condition. *x*-axis in F: GO flake counts, *y*-axis: base-10 logarithmic calculation of size (nm^2^).

Zeta potential is an important characteristic indicating the stability of colloidal dispersions. The low zeta potential values for the five samples, ranging from −5.53 ± 0.36 mV to −7.39 ± 0.56 mV, means a low stability of GOs in the SD medium. Moreover, the UV-treated and thermally reduced GOs have lower zeta potential than the other three, indicating that the former two samples are more inclined to aggregate in the SD medium. However, after adding the dispersant (1% BSA), the aggregation was quite slight, indicated by the similar aggregation size determined by the DLS of GOs prepared in the same way as for toxicogenomic assays. The Z-average size data also showed much smaller sizes for the two sonicated samples, which is consistent with the analysis by the SEM. As shown in the SEM image (Figure 1), the sonicated samples are much more scattered than in the other three samples. The size distribution, determined by image analysis, indicates an average surface area for untreated control 45-min-sonicated, and 2.5-h-sonicated GO samples of 5.31 ± 1.41 µm^2^, 0.70 ± 0.05 µm^2^, and 0.14 ± 0.007 µm^2^, respectively. The UV-treated and thermally reduced GO samples show a similar size distribution as the untreated control GO (Figure 1F and Table 1).

### 2.2. Distinctive Toxicity Profiles among Five GOs with Varying Physiochemical Properties Revealed by the Quantitative Toxicogenomic Analysis

**Figure 2:**
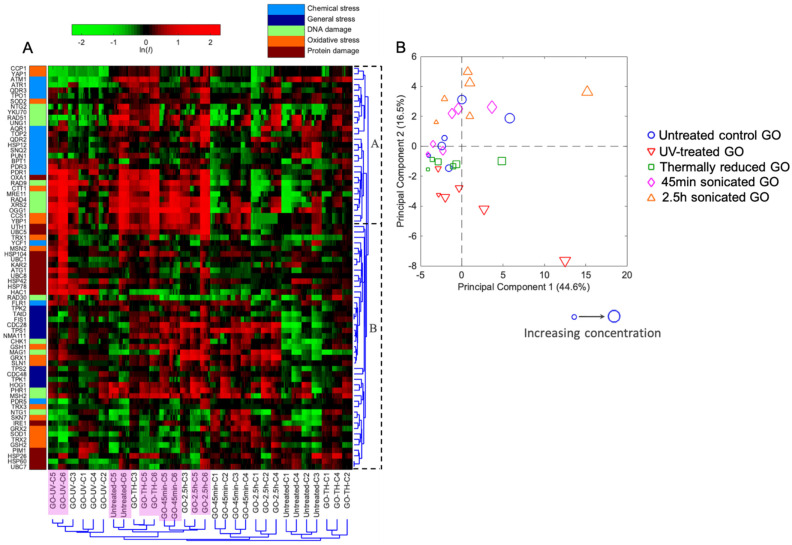
(**A**) Hierarchical cluster (HCL) analysis diagram on the basis of differential protein expressions (ln*I*, average of triplicates) of the 74 stress biomarkers in yeast in response to the five studied graphene oxides (GOs). The mean natural log of positive induction factors (ln*I*) indicates the magnitude of regulated protein expressions (scaled by the green-black-red color spectrum at the top. Green color spectrum indicates downregulation, and red color spectrum indicates upregulation. The ln*I* values beyond ±2 are indicated as ±2). *x*-axis bottom: sample names and concentrations of the GOs. C1-C6 indicate concentrations 1-6, which are 0.031, 0.125, 0.5, 2, 8 and 32 mg/L, respectively. Higher concentrations C5 and C6 are in purple background. *y*-axis **left**: list of proteins categorized within five stress categories (captions shown at top). *y*-axis **right**: cluster root of protein markers and subclusters. (**B**) Principal component analysis (PCA) with differential protein expressions (ln*I*, average of triplicates) in a GFP-fused yeast library exposed to the 5 GOs across 6 concentrations. Samples are color-coded and each legend shape indicates one treatment with a larger legend size representing the higher concentration.

A hierarchical cluster (HCL) analysis diagram, based on the ln*I* values of the 74 selected stress biomarkers in yeast in response to the five graphene oxides (GOs), was performed (Figure 2A). The results indicate distinctive protein expression profiles for the five GO samples that had undergone different treatments in a concentration-dependent manner. Generally, the GOs that had gone through the same treatment were clustered together at higher concentrations (32 and 8 mg/L, C6-5 purple-colored in Figure 2A), suggesting a treatment-associated similarity in their properties. Different GOs at lower concentrations (≤2 mg/L, C4-1 in Figure 2A) that were clustered together likely resulted from the overall indistinguishable low background-level toxicity (Figure 2A and SI Figure S1). The exception is the UV-treated GO, for which all six concentration samples were clustered together. This indicates that UV-treated GOs exhibit more distinctive toxicity profiles from other GOs. Thermally reduced GOs were clustered closely with untreated GOs, suggesting a nondramatic modification effect of the thermal reduction treatment.

From the clustering of differentially expressed protein marker patterns, we can identify the proteins sharing common regulation behaviors for biological pathways upon exposure to the five GOs. The proteins sharing a similar coregulation in the same stress category (e.g., chemical stress) were grouped into two subclusters, A and B (Figure 2A). Subcluster A mostly comprised proteins in the oxidative, chemical, and DNA damage stress categories, whereas Subcluster B generally involved biomarkers related to protein and general stress. The biomarkers in Subcluster A exhibited a generally higher level in differential protein expression upon the five GO treatments at the higher concentrations (32 and 8 mg/L), suggesting the high oxidative, chemical, and DNA damage impacts of the five GOs at higher, yet subcytotoxic, concentrations.

Principal component analysis (PCA) based on differential protein expression profiles in response to the five GOs across six concentrations showed that the first two principal components explained more than 60% of the total variance among all the samples (Figure 2B). The difference in toxicity profiles between low-concentration and high-concentration samples seemed to be captured by the first principal component. The results also reveal that untreated GO and GO that was subjected to different treatments (UV, thermal, and sonication), and those at varying concentrations, were separated with different projection directions, suggesting that they all have distinct toxicity profiles and underlying molecular mechanisms. Notably, the UV-treated and lengthier-timed 2.5-h-sonicated GOs exhibited the largest concentration-dependent variations along the first principal component, indicating the most distinct toxicity profile for these GO samples (Figure 2B).

### 2.3. Insights into Toxicity Mechanisms Revealed by Comparison among Various GOs

**Figure 3:**
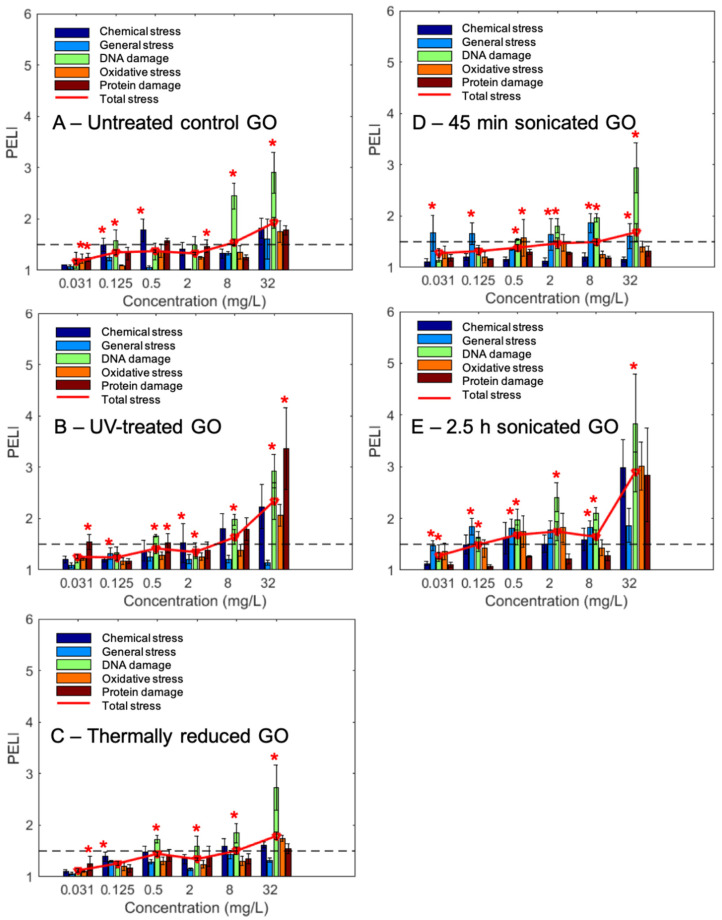
Toxicity profiles of the 5 studied graphene oxides (GOs) in terms of the 5 stress categories and the total protein expression level index (PELI): (**A**) untreated control GO; (**B**) GO treated with 96 h UV; (**C**) GO treated with 96 h thermal reduction; (**D**) GO treated with 45 min bath sonication; and (**E**) GO treated with 2.5 h bath sonication. Toxicity positive was defined as possessing a PELI value larger than a 1.5. *x*-axis: concentrations of examined GOs (mg/L). *y*-axis: PELI as molecular toxicity endpoint. Mean ± SD, replication number *n* = 3, * *p* < 0.05.

The quantitative comparison of the molecular toxicity level of the five GO samples, for each of the five stress categories (i.e., general, chemical, DNA, oxidative, and protein stress) are further compared in Figure 3. The PELI values for various stress categories across the concentration gradients for all five GO samples are also illustrated in Figure 3, where the specific treatments that had significant enrichment in certain toxicity categories are highlighted with an asterisk. The PELI values show concentration-dependent increasing trends for all GOs, although the magnitude of various categories varied among them. For all five samples at high concentrations, DNA stress toxicity generally stood out as the dominant toxicity effect. By comparison, both the thermally reduced GO, and the 45-min-sonicated GO, exhibited a similar toxicity trend and level to the untreated GO, even though there is a significant difference in the average size and size distribution (Z-average is 1461 nm for thermally reduced GO, and 446.6 nm for 45-min-sonicated GO) (Table 1). However, for the two size-modified GO samples, the 45-min-sonicated GO sample had a similar toxicity trend and level to the untreated GO, while the 2.5-h-sonicated GO exhibited a much higher toxicity level, with nearly all five toxicity categories exhibiting significantly higher PELI levels (*p* < 0.05) than the untreated GO. A size distribution analysis (Figure 1F) shows that extended sonication (2.5 h) shifted the size distribution significantly to a much smaller sized GO, which seemed to correspond to the elevated toxicity effects.

The PELI-based molecular endpoint PELI1.5 (mg/L) was determined from the 4PL nonlinear concentration-response curves (SI Figure S2) at the studied concentration range, i.e., 0.031–32 mg/L, the results of which are summarized in Table 2. Comparing the PELI1.5 totals, we can see that the overall toxicity levels of UV-treated, thermally reduced, and 45-min-sonicated GOs were comparable, and that the untreated control GO exhibited a lower toxicity level than the former three. The exact PELI1.5 total of the 2.5-h-sonicated GO cannot be generated from the fitted curve since it was above PELI = 1.5 at the examined concentration range (0.031–32 mg/L), indicating that the 2.5-h-sonicated GO probably had a PELI1.5 total of less than 0.031 mg/L and, thus, has the highest total toxicity level. Moreover, the toxic equivalents geno-TEQ1.5 and oxi-TEQ1.5, induced by each GO at PELI1.5, were calculated using MMC and H2O2 as reference compounds, respectively (Table 2). The results indicate that the untreated control GO possessed the lowest geno-TEQ1.5, and the 2.5-h-sonicated GO showed the highest genotoxic equivalent. In the aspect of oxidative stress oxi-TEQ1.5, the 45-min-sonicated and 2.5-h-sonicated GOs showed the lowest and the highest levels, respectively.

GSEA was performed to analyze the stress categories to further compare the toxicity mechanisms and responses among the five GOs (SI Table S2; Figure 3). The results show that DNA damage was the most frequently enriched stress category for all five GOs, especially in the high concentration range. For the two sonicated GO samples, in addition to DNA damage stress, general stress was also the dominant category with significant enrichment. For the untreated, UV-treated, and thermally reduced GOs, chemical stress and protein stress were also significantly indicated by the GSEA.

A gene ontology enrichment analysis was performed for the activated ORFs (PELIORF > 1.5) in yeast cells in response to the exposure of the five GOs at 32 mg/L, which had the most ORFs activated (SI Table S3). The DNA metabolic process, the response to DNA damage stimulus, and DNA repair were overrepresented (*p*-value < 0.05) for the untreated control GO and the UV-treated GO, suggesting DNA stress was one of the main stress categories, which agrees with the GSEA results. For the thermally reduced GO, the regulation of the protein metabolic process was significantly enriched. In response to 45-min- and 2.5-h-sonicated GO exposure, more gene ontological biological processes were overrepresented than in the previous three GOs. Cellular responses to stress and stimulus, and various DNA stress-related processes, such as DNA repair, damage stimulus, and metabolic processes were significantly enriched for both the 45-min- and 2.5-h-sonicated GOs. Additionally, the nitrogen compound metabolic process and cell cycle was only overrepresented for 45-min-sonicated GOs and 2.5-h-sonicated GO, respectively. Moreover, gene ontology cellular components’ intracellular part and nuclear part, the nucleus and the nuclear part, as well as the nucleus, were significantly represented in response to the UV-treated GO, the 45-min-sonicated GO, and the 2.5-h sonicated-GO, respectively, indicating that the nucleus/nuclear part might be one of the target sites for these three GOs.

### 2.4. Molecular Toxicity Endpoints Correlated with Conventional Phenotypic Toxicity Endpoints of GOs

**Figure 4:**
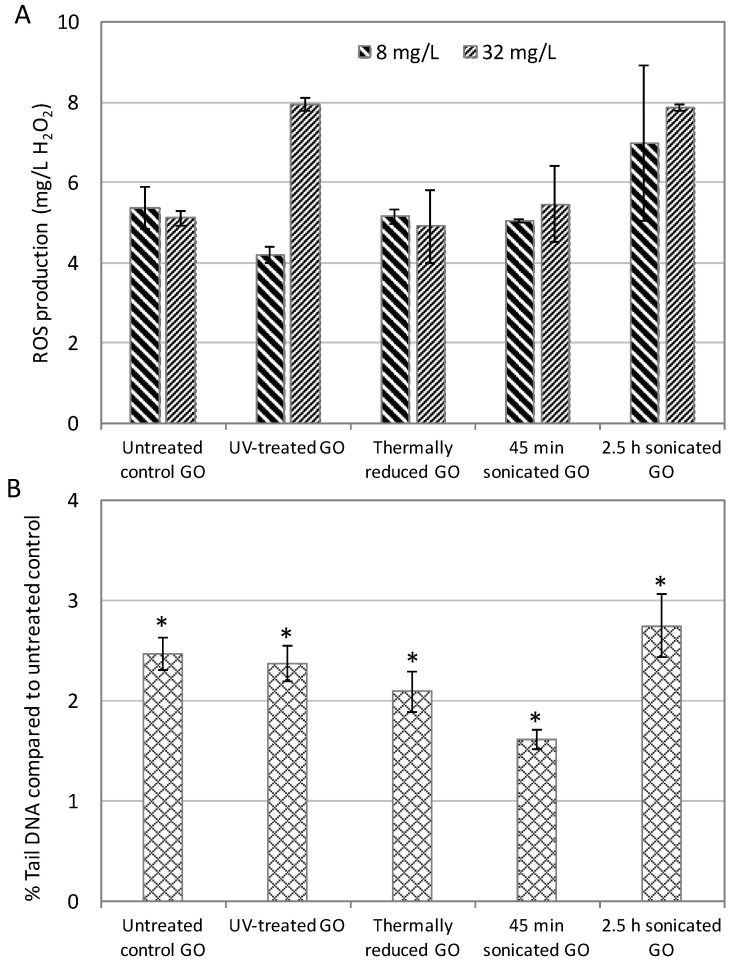
(**A**) Intracellular reactive oxygen species (ROS) production (equivalent to mg/L H_2_O_2_) in yeast cells induced by the 5 tested graphene oxides (GOs), and (**B**) % Tail DNA compared to the untreated control determined by the alkaline comet assay for the 5 GOs. The % Tail DNA compared to the untreated control indicates DNA damage caused in human A549 cells by the 5 GOs. (**A**) and (**B**): X-axis bottom: name of the 5 GOs; Y-axis: (**A**) ROS production (equivalent to mg/L H_2_O_2_), and (**B**) % Tail DNA compared to the untreated control. The “*” indicates a significant difference from the untreated control (*p* < 0.05). Mean ± SD. For ROS production, *n* = 3; for comet assay, *n* = 4.

**Figure 5:**
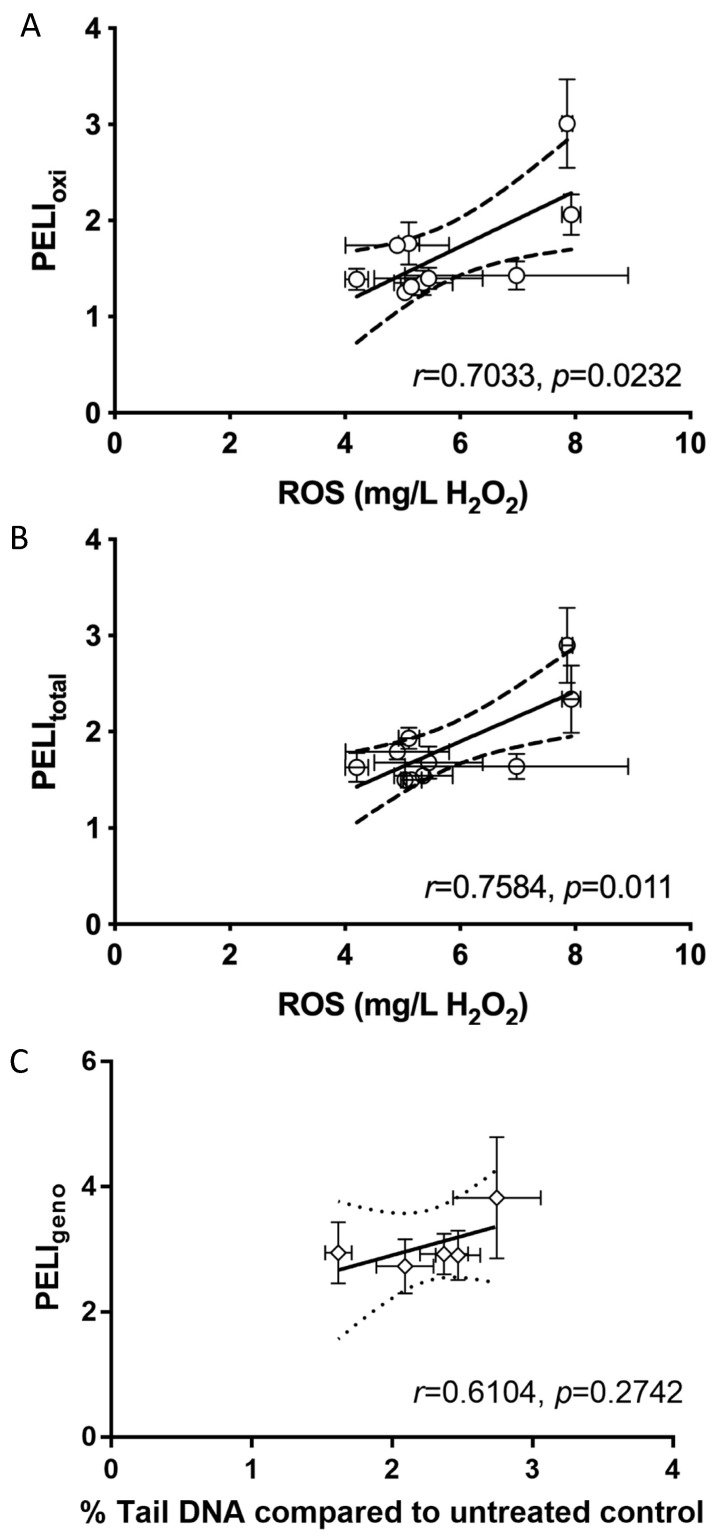
Correlation of intracellular ROS generation (equivalent to mg/L H_2_O_2_) with PELI_oxi_ (**A**) and PELI_total_ (**B**) in yeast, and correlation of % Tail DNA compared to the untreated control with PELI_geno_ (**C**) for the 5 tested graphene oxides (GOs). The % Tail DNA compared to the untreated control examined by the alkaline comet assay indicates DNA damage caused in human A549 cells by the 5 GOs. The 95% confidence intervals are indicated by the dash lines. (**A**) and (**B**): *x*-axis bottom: ROS production in yeast equivalent to mg/L H_2_O_2_; *y*-axis: PELI_oxi_ (**A**) or PELI_total_ (**B**) in yeast assay. (**C**) X-axis bottom: % Tail DNA compared to untreated control; Y-axis: PELI_geno_ in yeast assay. *r* indicates Pearson correlation coefficient; *p* value shows whether the correlation is significantly linear. Mean ± SD. For ROS production, *n* = 3; for comet assay, *n* = 4. An error bar was not shown when it was smaller than the marker size.

ROS are the reactive free radicals and molecules that are generated from molecular oxygen, which has been proposed as one of the toxicity mechanisms of GOs [50]. Using the conventional phenotypic bioassay, we examined the intracellular ROS production in yeast cells in response to the five GOs at the two higher concentrations (8 and 32 mg/L) (Figure 4A) and correlated the ROS production values with the oxidative and total PELI values (Figure 5A and B). The results show that the 2.5-h-sonicated GO induced the highest ROS production at 8 and 32 mg/L (Figure 4A). The correlation results show that both the oxidative stress PELI values and the total molecular toxicity PELI values had statistically strong correlations with ROS production. In addition, ROS production had a better correlation with total PELI values (*r* = 0.7584, *p* = 0.011) than oxidative PELI (*r* = 0.7033, *p* = 0.0232), indicating that ROS not only induced oxidative stress, but also might result in other stresses, such as DNA and protein stress. The strong correlation observed between the molecular and phenotypic endpoints elucidates that the quantitative molecular disturbance quantifiers, based on the regulated expressions of key protein markers related to oxidative stress pathways, can successfully capture the ROS production potential, thereby potentially quantitatively predicting phenotypic outcomes in terms of ROS production.

DNA damage was the main mode of the mechanisms for all five tested GOs. Therefore, we examined the DNA damage employing an alkaline comet assay, which is a conventional standard genotoxicity assay that has been commonly performed in human cells [51,52]. The phenotypic genotoxicity endpoint (% Tail DNA) based on the comet assay in human A549 cells in response to the five GOs was measured, and the results show that all five GOs exhibited positive genotoxicity (Figure 4B). The 2.5-h-sonicated GO appeared to induce the highest genotoxicity determined by the comet assay. The correlation between the DNA damage endpoints in the comet assay and the molecular genotoxicity endpoint PELI values were examined, and the results show that they were well-correlated (*r* = 0.6104; Figure 5C).

## 3. Discussion

### 3.1. Toxicity Mechanisms of the Untreated GO

Previous studies suggest that the toxicity of GO is mainly attributed to excessive ROS production, elevated oxidative stress, DNA damage, and apoptosis in eukaryotes, such as zebrafish and humans [53,54]. Our results suggest that genotoxicity related to DNA damage is the predominant toxicity effect of untreated GO (Figure 3). The concentrations we applied were all at the subcytotoxic level, which is consistent with the results from previous studies in which the sublethal concentrations (no detectable cytotoxicity) led to detected genotoxicity [44,48,55]. The protein markers related to all the DNA repair pathways were upregulated in response to the untreated GO exposure, indicating a broad spectrum of DNA damage and repair pathway activation induced by GO. The gene ontology analysis confirmed that the DNA metabolic process was overrepresented, and various proteins in all the DNA repair pathways were involved, such as PHR1 related to direct reversal repair (DRR), OGG1 related to base excision repair (BER), as well as XRS2 and MRE11 related to double strand break (DSB).

GO exposure has been demonstrated to induce cell wall destruction in white moss *L. glaucum* and human stem cells [56,57], as well as cell membrane damage in green algae *R. subcapitata* and mussel *M. galloprovincialis* [5,58]. Our protein expression analysis reveals that the expression of a plasma membrane protein with a role in cell wall integrity, PUN1, was upregulated, suggesting that the cell wall might be one of the target sites upon untreated GO exposure. In addition, the ATP-binding cassette (ABC) transporters, YCF1, SNQ2, and ATM1, as well as the major facilitator superfamily (MFS) transporters, AQR1, ATR1, TPO1, and TOP2 exhibited upregulated expressions, suggesting possible effects from the contact, or the possibly internalization of GO sheets by yeast cells. Furthermore, some vital ions, such as iron, could be transported outside the cells because of the strong binding of iron to oxygen-functional groups on the GO surface, leading to iron deficiency and inhibitory metabolism in eukaryotes (e.g., *S. cerevisiae*, *C. albicans*, and *K. pastoris*) [59].

### 3.2. Effect of UV Treatment and Thermal Reduction on Toxicity of GOs

It has been proven that UV treatment could induce the surface activation of GO by the photodesorption of adsorbed molecules (e.g., O_2_ and H_2_O) on GO [60]. In our study, compared to the untreated GO, the UV-treated GO possessed higher carboxyl (C-COOH) and carbon-carbon groups (C-C and C=C), lower epoxy (C-O-C) and hydroxyl (C-OH) functional groups, and similar oxygen content (Table 1). These features might contribute to the significantly higher molecular toxicity related to DNA damage stress, protein stress, and chemical stress. The correlation analysis between PELI1.5_total_ values (Table 2) with epoxy and hydroxyl groups (Table 1) indicates that they have a positive linear relation (*r* = 0.65), i.e., the GO with lower epoxy and hydroxyl groups tended to have a lower PELI1.5_total_ and, thus, a higher toxicity.

The toxicity of reduced GO has also been investigated in eukaryotes. It has been demonstrated that the reduced GO induces severe and long-lasting injury in the cells of humans and animals [61,62]. Du et al. studied reduced GO toxicity on algal cells, and summarized the modes of action that were similar to GO [63]. Initially, the reduced GO enveloped the algal cells by adhering to the cell surfaces. Then, it induced a perturbation of the cell wall and membrane integrity, as well as nuclear chromatin condensation. Lastly, it increased ROS and malondialdehyde (MAD) production and inhibited antioxidant systems, consequently inducing oxidative stress in algal cells. Despite the fact that the toxicity mechanisms of reduced GO in algal cells have been proposed as similar to GO, their extents of toxicity may be varied [63].

Numerous previous studies reveal contradictory conclusions regarding the comparison of toxicity between the reduced GO and the untreated control GO [30,58,63,64,65,66]. For example, Katsumiti et al. found that reduced GO functionalized with polyvinylpyrrolidone (PVP) (rGO-PVP) was more toxic than GO and GO-PVP in mussel due to the higher degree of internalization and ROS generation for rGO-PVP [58]. In contrast, Kang et al. demonstrated that GO had a more potent toxicological effect than reduced GO in neural pheochromocytoma-derived pc12 cell lines, with apoptosis and cell cycle arrest as the main toxicity pathways [64]. In fact, the reduction usually resulted in the smaller size of GO because the decomposition of oxygen-containing groups also removed carbon atoms from the carbon plane and split the GO sheets into smaller pieces [67]. Therefore, the size of GO and reduced GO compared to most previous studies was fairly different, and this confounded the effect of reduction [68]. Our findings indicate that the toxicity of thermally reduced GO and untreated GO with comparable sizes stayed similar, suggesting no significant impact on GO toxicity from thermal reduction treatment. The low extent of reduction may partially contribute to the similar toxicity.

### 3.3. Impact of Size on GO Toxicity

The effects of GO size on the distribution was investigated in mice in vivo [28], and the results suggest that small-sized and large-sized GO mainly accumulated in different organs of mice, indicating a size-dependent dispersion state of GO. A modeling investigation conducted by Mao et al. indicated that smaller-sized GO tended to adhere to the surfaces of cell membranes instead of penetrating lipid bilayers [69]. Perreault et al. studied the dimension-dependent antibacterial activity of GO using *E. coli* [26], and the results indicated that the antimicrobial activity of GO was elevated with the deceasing size on the coating surface, while it decreased with the decreasing size in suspension. Different mechanisms of toxicity were proposed for different systems, i.e., oxidative stress related to the higher defect density of smaller sheets on the coating surface, and cell inactivation by GO entrapment in suspension. Liu et al. investigated the impacts of the GO lateral size on toxicity in suspension using *E. coli* and generated a similar conclusion as Perreault et al. [29]. However, the toxicity mechanisms might be largely distinct when applied to different cell lines in vitro, especially eukaryotic cells that are more relevant to humans, where the relative size comparison might be different because of the larger dimensions of eukaryotic cells.

The obviously distinct toxicity patterns and levels can be observed between the untreated control and the size-reduced GOs. Specifically, as the surface area decreased from 5.31 ± 1.41 µm^2^ (untreated control GO) to 0.70 ± 0.05 µm^2^ (45-min-sonicated GO), the toxicity levels and protein marker expression patterns stayed similar. However, when the surface area was reduced even lower, to 0.14 ± 0.007 µm^2^ for 2.5-h-sonicated GO, the toxicity levels were elevated significantly higher in almost all five categories than in the untreated control GO. Perreault et al. studied the size-dependent antibacterial activity of GO using *E. coli* [26], and the results indicate that the antimicrobial activity of GO increased with deceasing size on the coating surface, while it decreased with decreasing size in suspension. Different mechanisms of toxicity were proposed for different systems, i.e., oxidative mechanisms related to the higher defect density of smaller sheets on the coating surface, and cell inactivation by GO entrapment in suspension. Nevertheless, the yeast cell line we used in our study has a much larger size than *E. coli*, with a size of 50 μm^2^ for yeast compared to 5 μm^2^ for *E. coli.* Therefore, in the suspension system in our study, it is impossible for the much smaller GO sheets to entrap yeast cells, and the covering and subsequent internalization of the GO sheets might be the main mode of action in yeast cells. For the large-sized GO (untreated control), the adhesion of GO to yeast cells, and the internalization of a very small portion of tiny GO particles, might be the first step for the manifestation of GO toxicity. The size of the 45-min-sonicated GOs is smaller than that of the untreated control one, but it is still not small enough for the easier internalization of GO by yeast cells, leading to a similar toxicity level between them. With respect to the small-sized GO (2.5 h sonicated), the internalization of tiny GO sheets would be the main mode of action. In addition, a much larger number of GO edges that are active in providing carbon-carbon and oxygen-contained carbon groups, could increase the ROS level of yeast cells.

## 4. Materials and Methods

### 4.1. Nanomaterials Information and Preparation

Graphene oxide (GO) was obtained in an aqueous solution (4 mg/mL) from Graphenea (Cambridge, MA, USA) and diluted in deionized (DI) water to 0.5 mg/mL. The thermally treated GO (GO-TH) was obtained by heating the GO solution in a water bath at 60 °C for 96 h. The GO treated with UV (GO-UV) was obtained by exposing the GO solution to UV light (power = 4 W; λ = 254 nm) in ambient air for 96 h. For both GO-TH and GO-UV, magnetic stirring was used to ensure a homogeneous reduction of GO. The GO treated with sonication was obtained by bath-sonicating the GO solution in a Branson sonicator (volume = 1.9 L, maximal power = 80 W, and frequency = 20 kHz) for a desired time (45 min or 2.5 h).

GO stock solutions were made with twenty times the highest examined concentrations (32 mg/L) in phosphate buffered saline (PBS) with the commonly used 1% bovine serum albumin (BSA; Acros, NJ, USA) as dispersant [70,71]. To disperse the stock solutions well, they were sonicated by a bath sonicator at ∼130 watt for 15 min. Subsequently, they were immediately diluted in a synthetic defined (SD) medium for the following experiments.

With the intent to perform an initial hazard assessment and screening that would further facilitate environmentally relevant risk assessment, and due to our toxicogenomic assay’s high sensitivity for attaining the subtle molecular response, we chose six subcytotoxic concentrations with a four-fold decrease from IC5 (5% inhibitory concentration in cytotoxicity test) as the highest subcytotoxic concentrations (i.e., 32, 8, 2, 0.5, 0.125, and 0.031 mg/L), similar to previous nanomaterial toxicogenomic research [44,48,49].

### 4.2. GO Characterization

The characterization of GOs was performed using dynamic light scattering (DLS), a scanning electron microscope (SEM), and X-ray photoelectron spectroscopy (XPS). A DLS analyzer (Malvern Zetasizer Nano ZS90, Malvern, Worcestershire, UK) was used to measure the aggregation size, zeta potential, and conductivity of GOs in an SD medium over 2 h, which was the same as the exposure time in the toxicogenomic assay. For the SEM analysis, GOs were drop-casted onto a silicon wafer and their images were acquired with a field emission SEM (FE-SEM) with an in-lens secondary electron (Zeiss ULTRA, Pleasanton, CA, USA). The working distance and acceleration voltage were 3–4 mm and 5 kV, respectively. The statistical SEM image analysis of the GO flake sizes was performed employing the ImageJ software, where >300 GO flakes were analyzed for each condition. GOs were drop-casted onto a polymer and analyzed using a Thermo Scientific K-Alpha XPS instrument (Waltham, MA, USA) with X-rays obtained by a 12-kV electron beam with a 400-mm spot size. The elemental composition was quantified using Thermo Scientific Advantage software. The instrumental errors of XPS for detecting atomic percentages were ±1%, with a detection limit of 0.1% [72]. Therefore, a nondetection indicated an element of <0.1% in the sample. More details on GO characterization and purity are described in Supplementary Information.

### 4.3. Toxicogenomic Assay and Quantitative Molecular Endpoint Derivation

The toxicogenomic assay with a GFP-fused yeast reporter library (*S. cerevisiae*) was performed, as described previously [44,45,46,47,48,49]. The yeast library covered 74 key biomarkers (SI Table S1) indicative of all known key toxicity pathways in the five main stress categories, namely, general, chemical, DNA, oxidative, and protein stress [73,74,75,76]. For plate normalization and correction from background signals with GOs, we set two controls with or without GOs, including internal control (with housekeeping PGK1 yeast) and blank control (without yeast) [45]. The vehicle control was set as PBS with a 1% BSA. GOs and controls were added in the 384-well plate to the desired concentrations, followed by measurements of yeast growth (OD_600_) and GFP signals (Ex/Em = 485/535 nm) by a micro plate reader every 5 min over a 2 h period. All assays were conducted in triplicate. The toxicogenomic data analysis for endpoints derivation, at both single biomarker and pathway levels, can be found in Supplementary Information.

The 4 parameter logistic (4PL) regression was employed to derive PELI-based concentration-response curves for each GO, and a PELI > 1.5 indicated positive toxicity [44,46,48]. The PELI-based molecular endpoint PELI1.5 (mg/L) was generated, as described previously [45,47]. Moreover, toxic equivalents geno-TEQ1.5 and oxi-TEQ1.5, which denote genotoxicity and oxidative stress induced by GOs at PELI1.5 [48], were determined with mitomycin C (MMC) and H_2_O_2_ as reference standard chemicals, respectively [77,78].

### 4.4. Intracellular ROS Production Measurement

The intracellular ROS generated in yeast triggered by each GO at 8 and 32 mg/L was determined with 2′,7′-Dichlorofluorescin diacetate (DCFDA, Sigma-Aldrich), following the manufacturer’s protocol and literature [79,80]. Briefly, wild type yeast (GFP-negative) was incubated in an SD medium to OD_600_ of ~0.2. Then DCFDA was added and the yeast was incubated for another 45 min in the dark, followed by collection, PBS-wash, and resuspension of the yeast in the SD medium. The culture with an OD_600_ between 0.3–0.4 was added in 96-well plates, followed by the addition of GOs with the tested concentrations. The fluorescence intensity (Ex/Em = 485/535 nm) was monitored by a micro plate reader over a 2 h exposure period. The fold changes in ROS production induced by GOs were derived and compared to the blank medium control (with DCFDA and without yeast), and the chemical (with DCFDA and without yeast), and stained controls [48]. In addition, the equivalents to the positive control H_2_O_2_ were determined. All assays were conducted in triplicate.

### 4.5. DNA Damage Alkaline Comet Assay in Human A549 Cells

The 24 h alkaline comet assay in human lung epithelial cells A549 (ATCC CCL-185, Manassas, VA, USA), treated with each GO at 100 mg/L, was conducted following the ITRC protocol [81] and literature [48]. The 1% FBS-F12 medium served as the untreated control. Twenty-five cells were randomly chosen for each GO treatment, and the DNA damages were determined as % Tail DNA, which was considered as genotoxicity positive if the % Tail DNA of treatment significantly (*p* < 0.05) increased in contrast with the untreated control. All assays were conducted in 4 replicates.

### 4.6. Data Analysis

The clustering of differentially expressed proteins in response to the 5 GOs across 6 concentrations was achieved through hierarchical cluster analysis (HCA) using MATLAB (R2020a). The complete average linkage clustering order based on the correlation distance unveiled the relations among the 30 treatments. The complex datasets of categories were simplified by principal component analysis (PCA) through the examination of the largest variance based on their differentially expressed protein profiles.

Moreover, gene set enrichment analysis (GSEA) was performed to assess the activity of each stress category by sorting the related protein set based on the PELI values [82]. The significance for each stress category was derived by comparing its ranking scores with empirical distributions. Gene ontology enrichment was conducted, following the network ontology analysis (NOA) approach [83], to determine the overrepresented (significantly enriched, *p* < 0.05) biological categories, i.e., biological processes, cellular components, and molecular functions. Gene ontology was analyzed for the treatments with the most activated proteins (ORFs) (PELI_ORF_ > 1.5) for each GO (i.e., at 32 mg/L), using the whole 74 biomarkers library as the reference, and activated ORFs as the test set [46].

## 5. Conclusions

In conclusion, the toxicity of graphene oxides (GOs), and its dependence on oxidation level, elemental composition, and size, were comprehensively and systematically evaluated with five GOs, i.e., untreated control GO, UV-treated GO with different elemental compositions, thermally reduced GO with a lower oxidation level, and two sonicated GOs with smaller sizes. The results show that elemental composition and size do indeed exert impacts on GO toxicity, while the oxidation level exhibited no significant effects. The UV-treated GO, with significantly higher carbon-carbon groups and carboxyl groups (C-COOH), showed a higher toxicity level, especially in the protein and chemical stress categories. With the decrease in size, the toxicity level of sonicated GOs tended to increase. We proposed that the covering and subsequent internalization of GO sheets might be the main mode of action to yeast cells.

The comprehensive and systematic evaluation on the toxicity profiling and mechanisms at the molecular level of untreated and treated GOs fills the knowledge gap on GO molecular toxicity and its dependence on various physicochemical characteristics. The derived high-resolution molecular fingerprint can also serve as a screening tool to feasibly guide GO preparation, treatment, and risk management. Furthermore, the generated data can direct the development of the prototypic quantitative structure-activity relationship (QSAR) model with hierarchic structures to predict GO toxicity, which integrates the current QSAR framework with bioassay data by correlating GO descriptors with toxicity endpoints at both the molecular and phenotypic levels.

## Figures and Tables

**Table 1:** Characterization of graphene oxides. Changes in zeta potential, conductivity, and aggregation size were measured in synthetic defined (SD) medium during a 2 h period.

GOs		Untreated Control	UV-Treated	Thermally Reduced	45 min Sonicated	2.5 h Sonicated
Oxygen content (%)		36.1 ± 0.1	35.0 ± 0.7	33.9 ± 0.1	36.1 ± 0.1	36.1 ± 0.1
O/C ratio (atomic)		0.60	0.57	0.55	0.60	0.60
Impurities		N < 1%, S = 2–3%	N < 1%, S = 2–3%	N < 1%, S = 2–3%	N < 1%, S = 2–3%	N < 1%, S = 2–3%
Monolayer (%)		>90	>90	>90	>90	>90
C-O-C and C-OH (%)		62 ± 2	51 ± 2	57 ± 2	62 ± 2	62 ± 2
C-C and C=C (%)		34 ± 2	42 ± 2	38 ± 2	34 ± 2	34 ± 2
C-COOH (%)		4 ± 2	7 ± 2	6 ± 2	4 ± 2	4 ± 2
Zeta potential (mV)		−7.19 ± 0.46	−5.46 ± 0.36	−5.53 ± 0.36	−6.11 ± 0.09	−7.39 ± 0.56
Conductivity (mS/cm)		10.21 ± 0.28	10.40 ± 0.29	10.23 ± 0.17	10.67 ± 0.09	9.72 ± 0.25
Size, Z-average (nm)	0 h	1331	1627	1461	447	348
	2 h	1610	1669	1479	471	352

**Table 2:** Summary of PELI-based molecular endpoint PELI1.5 (mg/L) and toxic equivalents geno-TEQ1.5 and oxi-TEQ1.5. PELI1.5 was determined from the four-parameter logistic (4PL) nonlinear concentration-response curves (SI Figure S3) at the studied concentration range for the 5 stress and total categories. Toxic equivalents geno-TEQ1.5 and oxi-TEQ1.5 are for genotoxicity and oxidative stress, respectively, induced by each GO at PELI1.5, using mitomycin C (MMC) and H2O2 as the reference compounds, respectively.

GOs	PELI1.5 (mg/L)						TEQ1.5	
	Chemical	General	DNA	Oxidative	Protein	Total	Geno-	Oxi-
Untreated control	0.13	9.17	2.09	16.01	0.16	6.06	0.34	0.21
UV-treated	1.71	N/A	1.42	12.13	6.32	4.48	0.51	0.28
Thermally reduced	1.47	N/A	0.98	18.09	N/A	4.85	0.73	0.19
45 min sonicated	N/A	<0.031	0.59	N/A	N/A	4.83	1.22	N/A
2.5 h sonicated	4.36	0.07	<0.031	<0.031	13.61	<0.031	>23.19	>109.77

Note: N/A: data not available. PELI1.5 is not available when the concentration-response curve falls below the line PELI = 1.5 at the highest studied concentration (32 mg/L).

## Data Availability

Derived data supporting the findings of this study are available from the corresponding author (A.Z.G.) on request.

## Supplementary Materials

The following are available online at https://www.mdpi.com/article/10.3390/ijms221910578/s1.
