# Supplementary material for: Dependence of Graphene Oxide (GO) Toxicity on Oxidation Level, Elemental Composition, and Size
Source: Int J Mol Sci. 2021 Sep 30;22(19):10578. doi: 10.3390/ijms221910578 (PMC8508828; doi:10.3390/ijms221910578)
Supplement: Supplementary file 1 [file ijms-22-10578-s001.zip › ijms-1377898-supplementary.pdf]

Supplementary Information for

Dependence of Graphene Oxide (GO) Toxicity on Oxidation Level, Elemental Composition and Size

Tao Jiang [1], Carlo Alberto Amadei [2], Yishan Lin [1, 3 *], Na Gou [1, 4], Sheikh Mokhlesur Rahman [1, 5], Jiaqi Lan [1, 6], Chad D. Vecitis [2], April Z. Gu [4 *]

1. Department of Civil and Environmental Engineering, Northeastern University, 360 Huntington Ave, Boston, MA 02115

2. John A. Paulson School of Engineering and Applied Sciences, Harvard University, Cambridge, MA 02138

3. State Key Laboratory of Pollution Control & Resource Reuse, School of the Environment, Nanjing University, Nanjing, China

4. School of Civil and Environmental Engineering, Cornell University, 220 Hollister Dr., Ithaca, NY 14853

5. Department of Civil Engineering, Bangladesh University of Engineering and Technology, BUET Central Road, Dhaka 1000, Bangladesh

6. Institute of Materia Medica, Chinese Academy of Medical Sciences and Peking Union Medical College, Beijing, 100050, China

* Corresponding author: aprilgu@cornell.edu; yshanlisa@hotmail.com

Table S1. Selected GFP-fused yeast reporter library of an assembly of proteins involved in different known cellular stress response pathways.

Stress	Function	Pathway		Protein involved
General stress response	Metabolism	Trehalose synthesis		TPS1, TPS2
		Osmotic stress		HOG1
	General function	Signal transduction		TPK1, TPK2, CDC28
		Apoptosis		CDC48, NMA111, Tat-D, FIS1
Oxidative stress	Sensor/regulator	Yap1p regulation<br>Skn7p regulation<br>Msn2p regulation		YAP1, YBP1<br>SKN7, SLN1<br>MSN2
	Defense system	Glutathione/ Glutaredoxin	Glutathione<br>Glutaredoxin	GSH1, GSH2<br>GRX1, GRX2
		Thioredoxin	Thioredoxin	TRX1, TRX2, TRX3
		Enzymatic system	SOD<br>Catalase<br>CYC related	SOD1, SOD2, CCS1<br>CTT1<br>CCP1
Chemical stress	Membrane, cell wall and cell structure			PUN1, HSP12
	ATP-binding cassette (ABC) transporters			PDR1, PDR3, YCF1, PDR5, SNQ2, BPT1, ATM1
	Major facilitator superfamily (MFS) transporters			FLR1, QDR2, ATR1, TPO1, AQR1, QDR3, TOP2
Protein stress	Cytosolic unfolded protein response (cytUPR)	Molecular chaperones		HSP104, HSP42, HSP78, HSP26
		Ubiquitin-proteasome system		UBC5, UBC8
	Endoplasmic reticulum UPR (erUPR)	Sensor/regulator		IRE1, HAC1
		Molecular chaperones		KAR2
		ER-associated protein degradation (ERAD)		UBC1, UBC7
	Mitochondrial UPR (mtUPR)	Molecular chaperones		HSP60, HSP78
		Proteolytic system		OXA1, PIM1
	Autophagy			ATG1, UTH1
DNA stress	DNA repair	DNA damage signaling (DDS)		CHK1, RAD9
		Direct reversal repair (DRR)		PHR1
		Translesion synthesis (TLS)		RAD30
		Base excision repair (BER)		OGG1, NTG1, NTG2, UNG1, MAG1
		Nucleotide excision repair (NER)		RAD4

		Mismatch repair (MMR)	MSH2
		Double strand break (DSB)	XRS2, MRE11
		Homologous recombination (HR)	RAD51
		Non-homologous end joining (NHEJ)	YKU70

Toxicogenomics Assay Data Processing

Details of data processing for the yeast toxicogenomics assay were described in our previous studies [1-5]. Briefly, OD and GFP raw data from the measurements were firstly corrected for any interference due to the media by subtracting the OD and GFP of the well with the blank control. The corrected OD and GFP signals are referred to as $OD_{corrected}$ and $GFP_{corrected}$, respectively. The corrected GFP signal ($GFP_{corrected}$) was then normalized to the cell numbers in the well ($OD_{corrected}$) as $P = GFP_{corrected}/OD_{corrected}$. The P values was then corrected for the vehicle internal control (housekeeping gene PGK1 with medium control on each plate) to normalize different plates among the replicates, which is denoted as Q. The altered expression level of a given protein at each time point because of nanomaterial exposure (termed as induction factor, *I*) is defined as $I = Q_{experiment}/Q_{control}$; where $Q_{experiment}$ is the internal control (PGK1) normalized P in experimental conditions with chemical exposure, and $Q_{control}$ is the internal control normalized P in the vehicle control conditions without any nanomaterial exposure. The induction factor, $I < 1$ indicates downregulation and the $I > 1$ indicates the upregulation of the biomarkers due to the chemical exposure. The Protein Expression Level Index, PELI, was calculated to aggregate the time series altered expression into a quantitative toxicity index. The chemical induced PELI of the protein was calculated as,

$$PELI_{ORF,i} = \frac{\int_{t=0}^{t} I dt}{T}; where\ I = 1, if\ I \leq 1, else\ I = I.$$

Where, t is the exposure time in every time-step and T is the total exposure time.

The pathway activation responses were obtained by integrating the expression changes of all tested protein markers (ORFs) in a pathway as,

$$PELI_{pathway,j} = \frac{\sum_{i=1}^{n} w_i \times \mathrm{PELI}_{ORF,i}}{n}$$

Where, n is the number of ORFs in a particular pathway, and w_i is the weight factor of ORFs. The value of 1 was assigned for all w_i in this study.

The PELI values of the 5 main stress categories, i.e. general, chemical, DNA, oxidative and protein stress, are the average of PELI of all pathways in the corresponded stress category.

References:

1. Lan, J., Gou, N., Gao, C., He, M., & Gu, A. Z. (2014). Comparative and mechanistic genotoxicity assessment of nanomaterials via a quantitative toxicogenomics approach across multiple species. Environmental Science & Technology, 48 (21): 12937-12945.

2. Lan, J., Hu, M., Gao, C., Alshawabkeh, A., & Gu, A. Z. (2015). Toxicity assessment of 4-methyl-1-cyclohexanemethanol and its metabolites in response to a recent chemical spill in west virginia, USA. Environmental Science & Technology, 49 (10): 6284-6293.

3. Lan, J., Gou, N., Rahman, S. M., Gao, C., He, M., & Gu, A. Z. (2016). A quantitative toxicogenomics assay for high-throughput and mechanistic genotoxicity assessment and screening of environmental pollutants. Environmental Science & Technology, 50 (6): 3202-3214.

4. Jiang T., Amadei C. A., Gou N., Lin Y., Lan J., Vecitis C. D., & Gu, A. Z. (2020). Toxicity of single-walled carbon nanotubes (SWCNTs): effect of lengths, functional

groups and electronic structures revealed by a quantitative toxicogenomics assay. Environmental Science: Nano, 7 (5): 1348-1364.

5. Jiang, T., Lin, Y., Amadei, C.A., Gou, N., Rahman, S.M., Lan, J., Vecitis, C.D., & Gu, A. Z. (2021). Comparative and mechanistic toxicity assessment of structure-dependent toxicity of carbon-based nanomaterials. Journal of Hazardous Materials, 418: 126282.

Characterization and Purity of Nanomaterials

The untreated control graphene oxide (GO) was characterized by the manufacturer in detail and raw data can be found in their technical datasheets. The untreated control GO was further purified by first heating to 300 °C in an oven for 4 h to remove amorphous carbon. Raman spectroscopy gave a G/D peak ratio of > 2 indicating minimal amorphous carbon impurities. The GO was then dispersed by bath sonication in concentrated HCl and heated to 60 ^{o}C overnight to remove any residual metals. The XPS signal-to-noise ratio was 0.1%, thus a non-detection indicates that an element is < 0.1% in the sample.

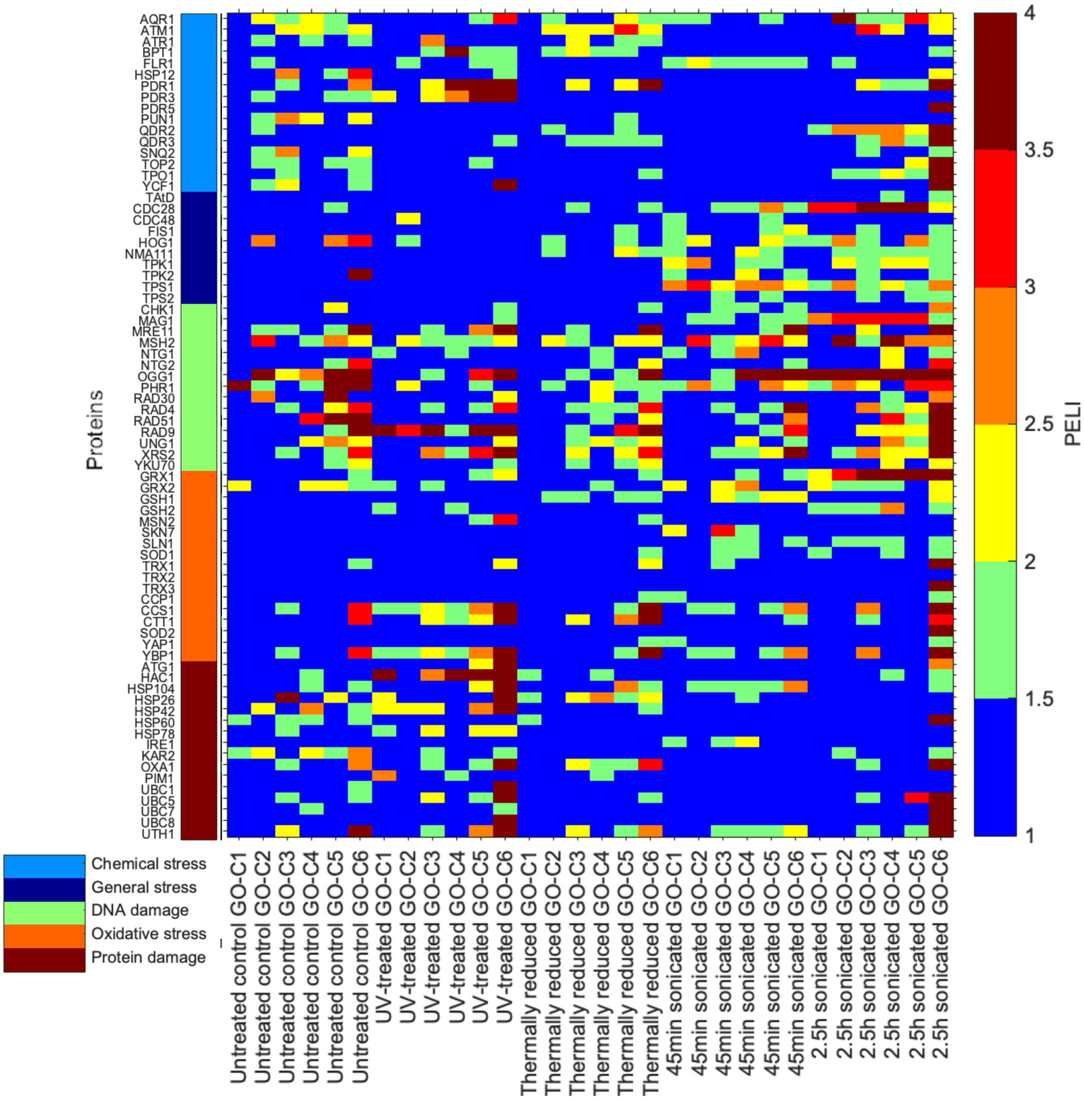

Figure S1. Protein expression profiles on the basis of PELI values of the 74 stress biomarkers in yeast in response to the 5 studied graphene oxides (GOs). X-axis bottom: sample names and concentrations of the GOs. C1-C6 indicate concentrations 1-6, which are 0.031, 0.125, 0.5, 2, 8 and 32 mg/L, respectively. Y-axis left: list of proteins categorized within five stress categories (captions shown at bottom). Y-axis right: scale of PELI values. The PELI values beyond 4 were indicated as 4. All tests were performed in triplicate (n = 3).

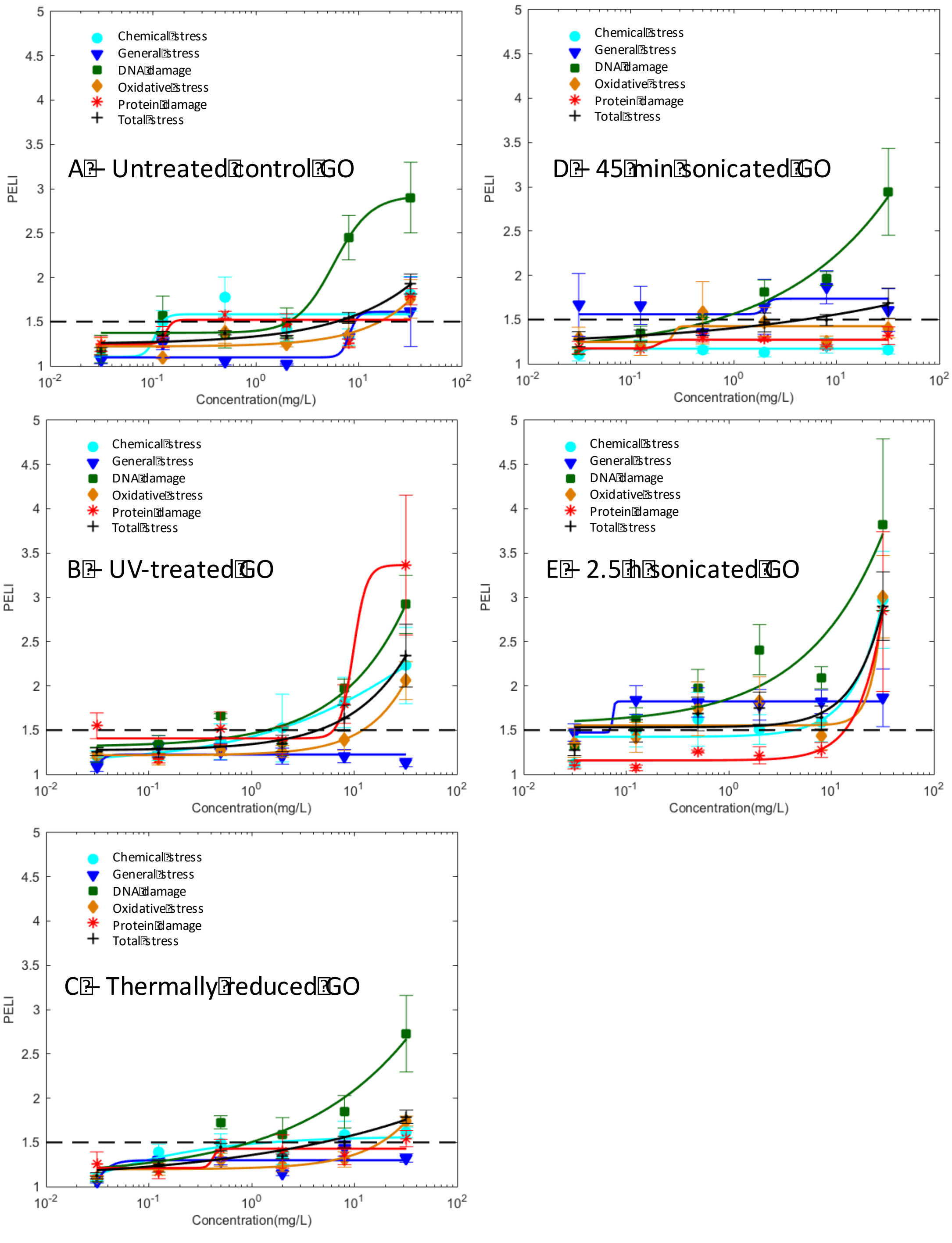

Figure S2. Concentration-response curves on the basis of PELI values of the 5 stress categories and total PELI for the 5 studied graphene oxides (GOs): (A) untreated control GO; (B) GO treated with 96 h UV; (C) GO treated with 96 h thermal reduction; (D) GO treated with 45 min bath sonication; and (E) GO treated with 2.5 h bath sonication. The curves were generated by Four Parameter Logistic (4PL) nonlinear regression model. Data points with error bars indicate the PELI values measured at each concentration.

Toxicity positive was defined as possessing a PELI value larger than 1.5. X-axis: concentrations of examined GOs (mg/L). Y-axis: PELI. Mean ± SD, n = 3.

Table S2. Summary of GSEA results for the 5 studied GOs based on toxicogenomics assay in yeast cells.

**Stress category**	**Protein set size**	**Enrichment score (ES)**	**Normalized enrichment score (NES)**	**Normal *p*-value**	**FDR-q value [a]**
		**Untreated control GO at 0.031 mg/L**			
**Protein**	15	-0.49	-1.82	0.01	0.03
**Oxidative**	16	-0.35	-1.31	0.17	0.26
**Chemical**	15	-0.22	-0.83	0.67	0.66
		**Untreated control GO at 0.125 mg/L**			
**Chemical**	15	-0.60	-1.97	0.00	0.01
**DNA**	17	-0.41	-1.43	0.08	0.14
**Protein**	15	-0.24	-0.80	0.69	0.71
		**Untreated control GO at 0.5 mg/L**			
**Chemical**	15	-0.58	-1.86	0.01	0.02
**Protein**	15	-0.37	-1.17	0.27	0.58
**DNA**	17	-0.18	-0.60	0.90	1.00
**Oxidative**	16	-0.16	-0.52	0.96	0.96
		**Untreated control GO at 2 mg/L**			
**Protein**	15	-0.47	-1.59	0.06	0.14
**Chemical**	15	-0.39	-1.31	0.16	0.26
**DNA**	17	-0.34	-1.19	0.27	0.27
		**Untreated control GO at 8 mg/L**			
**DNA**	17	-0.73	-2.25	0.00	0.00
**General**	9	-0.20	-0.51	0.96	0.96
		**Untreated control GO at 32 mg/L**			
**DNA**	17	-0.70	-2.11	0.00	0.00
**Protein**	15	-0.29	-0.87	0.64	1.00
**Chemical**	15	-0.30	-0.87	0.64	0.85
**Oxidative**	16	-0.18	-0.55	0.95	0.95
		**UV-treated GO at 0.031 mg/L**			
**Protein**	15	-0.53	-1.83	0.01	0.02
**Chemical**	15	-0.25	-0.86	0.61	0.93
**Oxidative**	16	-0.20	-0.72	0.77	0.78
		**UV-treated GO at 0.125 mg/L**			
**General**	9	-0.47	-1.44	0.10	0.30
**DNA**	17	-0.31	-1.18	0.28	0.42
**Chemical**	15	-0.32	-1.15	0.30	0.30
		**UV-treated GO at 0.5 mg/L**			

**DNA**	17	-0.43	-1.49	0.07	0.31
**Protein**	15	-0.37	-1.25	0.22	0.44
**General**	9	-0.40	-1.18	0.28	0.36
**Chemical**	15	-0.22	-0.77	0.74	0.73
**UV-treated GO at 2 mg/L**					
**Chemical**	15	-0.44	-1.49	0.07	0.30
**DNA**	17	-0.41	-1.44	0.10	0.19
**Oxidative**	16	-0.22	-0.77	0.72	0.98
**Protein**	15	-0.20	-0.67	0.84	0.86
**UV-treated GO at 8 mg/L**					
**DNA**	17	-0.49	-1.54	0.05	0.15
**Chemical**	15	-0.38	-1.14	0.32	0.47
**Protein**	15	-0.33	-0.98	0.49	0.49
**UV-treated GO at 32 mg/L**					
**Protein**	15	-0.62	-1.60	0.02	0.06
**DNA**	17	-0.53	-1.35	0.11	0.20
**Oxidative**	16	-0.30	-0.76	0.80	1.00
**Chemical**	15	-0.28	-0.72	0.83	0.83
**Thermally reduced GO at 0.031 mg/L**					
**Protein**	15	-0.52	-2.05	0.01	0.01
**Oxidative**	16	-0.18	-0.73	0.78	0.79
**Thermally reduced GO at 0.125 mg/L**					
**Chemical**	15	-0.34	-1.32	0.16	0.49
**General**	9	-0.36	-1.14	0.29	0.44
**DNA**	17	-0.15	-0.59	0.92	0.93
**Thermally reduced GO at 0.5 mg/L**					
**DNA**	17	-0.59	-2.14	0.00	0.00
**Chemical**	15	-0.30	-1.10	0.34	0.52
**Protein**	15	-0.27	-0.99	0.47	0.48
**Thermally reduced GO at 2 mg/L**					
**DNA**	17	-0.55	-2.10	0.00	0.00
**Protein**	15	-0.30	-1.09	0.36	0.54
**Chemical**	15	-0.12	-0.45	0.98	0.99
**Thermally reduced GO at 8 mg/L**					
**DNA**	17	-0.50	-1.79	0.02	0.04
**Chemical**	15	-0.24	-0.83	0.68	1.00
**General**	9	-0.23	-0.68	0.84	0.83
**Thermally reduced GO at 32 mg/L**					
**DNA**	17	-0.71	-2.16	0.00	0.00
**Oxidative**	16	-0.22	-0.65	0.87	1.00
**Chemical**	15	-0.13	-0.37	1.00	1.00
**45 min sonicated GO at 0.031 mg/L**					
**General**	9	-0.74	-2.26	0.00	0.00
**Oxidative**	16	-0.33	-1.19	0.26	0.39
**Protein**	15	-0.27	-0.95	0.50	0.51

**45 min sonicated GO at 0.125 mg/L**					
**General**	9	-0.59	-1.83	0.01	0.04
**DNA**	17	-0.26	-0.97	0.48	0.98
**Oxidative**	16	-0.26	-0.92	0.56	0.74
**Protein**	15	-0.24	-0.86	0.61	0.63
**45 min sonicated GO at 0.5 mg/L**					
**DNA**	17	-0.52	-1.94	0.01	0.03
**Oxidative**	16	-0.35	-1.30	0.18	0.33
**General**	9	-0.29	-0.87	0.64	0.83
**Protein**	15	-0.12	-0.42	0.99	0.99
**45 min sonicated GO at 2 mg/L**					
**General**	9	-0.50	-1.44	0.10	0.29
**DNA**	17	-0.40	-1.38	0.13	0.20
**Oxidative**	16	-0.32	-1.09	0.35	0.36
**45 min sonicated GO at 8 mg/L**					
**General**	9	-0.74	-2.04	0.00	0.00
**DNA**	17	-0.58	-1.98	0.00	0.00
**45 min sonicated GO at 32 mg/L**					
**DNA**	17	-0.76	-2.26	0.00	0.00
**General**	9	-0.50	-1.30	0.17	0.17
**2.5 h sonicated GO at 0.031 mg/L**					
**General**	9	-0.54	-1.58	0.05	0.15
**Oxidative**	16	-0.36	-1.26	0.20	0.32
**DNA**	17	-0.27	-0.95	0.53	0.52
**2.5 h sonicated GO at 0.125 mg/L**					
**General**	9	-0.54	-1.50	0.07	0.26
**DNA**	17	-0.43	-1.38	0.13	0.25
**Chemical**	15	-0.31	-0.98	0.52	0.66
**Oxidative**	16	-0.22	-0.71	0.81	0.80
**2.5 h sonicated GO at 0.5 mg/L**					
**DNA**	17	-0.58	-1.93	0.00	0.01
**General**	9	-0.53	-1.47	0.09	0.16
**Oxidative**	16	-0.25	-0.83	0.69	0.90
**Chemical**	15	-0.21	-0.68	0.84	0.84
**2.5 h sonicated GO at 2 mg/L**					
**DNA**	17	-0.60	-1.88	0.00	0.00
**General**	9	-0.35	-0.91	0.59	0.88
**Oxidative**	16	-0.22	-0.66	0.87	0.87
**2.5 h sonicated GO at 8 mg/L**					
**DNA**	17	-0.55	-1.70	0.02	0.04
**General**	9	-0.48	-1.28	0.19	0.29
**Chemical**	15	-0.28	-0.84	0.64	0.66
**2.5 h sonicated GO at 32 mg/L**					
**DNA**	17	-0.53	-1.55	0.03	0.11
**Protein**	15	-0.38	-1.09	0.38	0.76

**Chemical**	15	-0.36	-1.02	0.46	0.62
**Oxidative**	16	-0.31	-0.91	0.60	0.60

Note: a FDR: false discovery rate, indicative of probability that a protein set with a given NES represents a false positive finding. An enrichment with FDR < 25% is defined as significant based on GSEA, and FDR of 25% indicates that the result is likely to be valid 3 out of 4 times.

Table S3. Overrepresented (p-value < 0.05) biological categories based on gene ontology databases for a 2 h exposure with the 5 GOs at 32 mg/L in yeast cells, employing the selected stress library as the reference set.

Category	*p*-value	Names of observed markers in category [a]	Number of markers annotated by given term in reference set	Number of makers annotated by given term in test set
		**Untreated control GO at 32 mg/L**		
		**Gene Ontology Biological Processes**		
**DNA metabolic process [GO:0006259]**	0.0208	OGG1; TOP2; RAD4; XRS2; RAD9; MRE11; RAD51; UNG1; PHR1; NTG2; MSH2; CHK1; RAD30; YKU70; MAG1; NTG1	16	16
**response to DNA damage stimulus [GO:0006974]**	0.0274	OGG1; RAD4; XRS2; RAD9; MRE11; RAD51; UNG1; PHR1; NTG2; MSH2; CHK1; RAD30; YKU70; MAG1; NTG1	15	15
**DNA repair [GO:0006281]**	0.0359	OGG1; RAD4; XRS2; RAD9; MRE11; RAD51; UNG1; PHR1; NTG2; MSH2; RAD30; YKU70; MAG1; NTG1	14	14
		**Gene Ontology Molecular Functions**		
**catalytic activity [GO:0003824]**	0.0358	OGG1; UBC5; TOP2; MRE11; TRX1; RAD51; SOD2; HSP60; YCF1; UBC7; TRX3; UBC8; PDR5; UNG1; GRX1; PHR1; CTT1; NTG2; MSH2; TPS1; CHK1; ATG1; RAD30; YKU70; GRX2; CDC28; GSH1; ATM1; NMA111; TPK2; SLN1; TPK1; MAG1; NTG1; CCP1; KAR2; SOD1; TPS2; HOG1; HSP104; GSH2; SNQ2; BPT1	49	43
		**UV-treated GO at 32 mg/L**		
		**Gene Ontology Biological Processes**		
**DNA recombination [GO:0006310]**	0.0136	MRE11; RAD51; XRS2; YKU70; MSH2; TOP2	6	6

**DNA repair [GO:0006281]**	0.02	OGG1; PHR1; MRE11; RAD51; RAD9; RAD4; XRS2; NTG2; UNG1; YKU70; MSH2	14	11
**DNA metabolic process [GO:0006259]**	0.0263	OGG1; PHR1; MRE11; RAD51; RAD9; RAD4; XRS2; NTG2; UNG1; YKU70; MSH2; TOP2	16	12
**response to DNA damage stimulus [GO:0006974]**	0.0454	OGG1; PHR1; MRE11; RAD51; RAD9; RAD4; XRS2; NTG2; UNG1; YKU70; MSH2	15	11
		**Gene Ontology Cellular Components**		
**intracellular part [GO:0044424]**	0.0051	OGG1; PHR1; MRE11; TPK2; RAD51; UTH1; RAD9; RAD4; XRS2; HSP12; NTG2; CTT1; YBP1; CCS1; HOG1; PDR1; KAR2; OXA1; ATM1; UNG1; PUN1; YKU70; MSH2; SNQ2; YCF1; HSP60; PDR3; GRX2; HSP104; UBC5; TRX1; HSP42; TPO1; UBC1; TOP2; ATR1; GRX1	66	37
**nuclear part [GO:0044428]**	0.0304	MRE11; RAD51; RAD4; XRS2; YKU70; MSH2; TOP2	8	7
		**Gene Ontology Molecular Functions**		
**structure-specific DNA binding [GO:0043566]**	0.0136	MRE11; RAD51; RAD9; XRS2; MSH2; HSP60	6	6
		**Thermally reduced GO at 32 mg/L**		
		**Gene Ontology Biological Processes**		
**regulation of protein metabolic process [GO:0051246]**	0.0334	UBC1; UBC5; UBC8; HSP78; UBC7	5	5
		**45 min sonicated GO at 32 mg/L**		
		**Gene Ontology Biological Processes**		
**response to DNA damage stimulus [GO:0006974]**	0.0017	OGG1; MRE11; RAD9; RAD4; XRS2; MSH2; RAD51; YKU70; UNG1; NTG2; PHR1; CHK1; RAD30	15	13
**DNA repair [GO:0006281]**	0.0035	OGG1; MRE11; RAD9; RAD4; XRS2; MSH2; RAD51; YKU70; UNG1; NTG2; PHR1; RAD30	14	12
**DNA metabolic process [GO:0006259]**	0.0056	OGG1; MRE11; RAD9; RAD4; XRS2; MSH2; RAD51; YKU70; UNG1; NTG2; PHR1; CHK1; RAD30	16	13
**cellular response to stress [GO:0033554]**	0.0222	OGG1; CTT1; MRE11; RAD9; YBP1; RAD4; XRS2; TRX1; MSH2; RAD51; YKU70; UNG1; NTG2; PHR1; YAP1; CCP1; CHK1; NMA111; RAD30; HSP104; GRX1; MSN2	34	22

**cellular response to stimulus [GO:0051716]**	0.0222	OGG1; CTT1; MRE11; RAD9; YBP1; RAD4; XRS2; TRX1; MSH2; RAD51; YKU70; UNG1; NTG2; PHR1; YAP1; CCP1; CHK1; NMA111; RAD30; HSP104; GRX1; MSN2	34	22
**nitrogen compound metabolic process [GO:0006807]**	0.0316	OGG1; PDR1; MRE11; RAD9; RAD4; XRS2; TRX1; MSH2; RAD51; YKU70; UNG1; NTG2; PHR1; YAP1; CHK1; RAD30; ATR1; MSN2	27	18
**response to stress [GO:0006950]**	0.0356	OGG1; CTT1; MRE11; RAD9; YBP1; RAD4; XRS2; UTH1; TRX1; MSH2; RAD51; YKU70; UNG1; HSP26; NTG2; GSH1; UBC5; PHR1; YAP1; CCP1; CHK1; NMA111; RAD30; HSP104; GRX1; SOD1; HSP42; MSN2	47	28
**nucleic acid metabolic process [GO:0090304]**	0.0476	OGG1; PDR1; MRE11; RAD9; RAD4; XRS2; MSH2; RAD51; YKU70; UNG1; NTG2; PHR1; YAP1; CHK1; RAD30; MSN2	24	16
		**Gene Ontology Cellular Components**		
**nucleus [GO:0005634]**	0.0127	OGG1; PDR1; MRE11; RAD9; CCS1; RAD4; XRS2; TRX1; MSH2; RAD51; YKU70; UNG1; HSP26; NTG2; PHR1; YAP1; CHK1; CDC28; NMA111; RAD30; HSP104; GRX1; SOD1; MSN2	37	24
**nuclear part [GO:0044428]**	0.0304	MRE11; RAD4; XRS2; MSH2; RAD51; YKU70; YAP1	8	7
		**Gene Ontology Molecular Functions**		
**damaged DNA binding [GO:0003684]**	0.0029	OGG1; RAD4; RAD51; YKU70; RAD30	5	5
		**2.5 h sonicated GO at 32 mg/L**		
		**Gene Ontology Biological Processes**		
**response to DNA damage stimulus [GO:0006974]**	1.1×10^{-4}	OGG1; XRS2; RAD4; MRE11; RAD9; RAD51; PHR1; MSH2; CHK1; UNG1; MAG1; NTG2	15	12
**DNA metabolic process [GO:0006259]**	3.6×10^{-4}	OGG1; XRS2; RAD4; MRE11; RAD9; RAD51; PHR1; MSH2; CHK1; UNG1; MAG1; NTG2	16	12
**DNA repair [GO:0006281]**	3.6×10^{-4}	OGG1; XRS2; RAD4; MRE11; RAD9; RAD51; PHR1; MSH2; UNG1; MAG1; NTG2	14	11
**cellular response to**	0.0039	OGG1; XRS2; RAD4; MRE11; RAD9; YBP1; RAD51;	34	18

**stress [GO:0033554]**		HSP104; PHR1; MSH2; CHK1; CTT1; UNG1; MAG1; NTG2; GRX1; HOG1; SLN1		
**cellular response to stimulus [GO:0051716]**	0.0039	OGG1; XRS2; RAD4; MRE11; RAD9; YBP1; RAD51; HSP104; PHR1; MSH2; CHK1; CTT1; UNG1; MAG1; NTG2; GRX1; HOG1; SLN1	34	18
**base-excision repair [GO:0006284]**	0.0070	OGG1; XRS2; MRE11; UNG1; MAG1; NTG2	7	6
**DNA catabolic process [GO:0006308]**	0.0195	OGG1; MRE11; MSH2; MAG1; NTG2	6	5
**nucleic acid metabolic process [GO:0090304]**	0.0205	OGG1; XRS2; RAD4; MRE11; RAD9; RAD51; PHR1; MSH2; CHK1; UNG1; MAG1; NTG2; HOG1	24	13
**cell cycle [GO:0007049]**	0.0209	MRE11; RAD9; RAD51; UTH1; CHK1; CDC28	8	6
**response to stress [GO:0006950]**	0.0255	OGG1; XRS2; RAD4; MRE11; RAD9; YBP1; RAD51; HSP104; GSH1; TPS1; PHR1; MSH2; UTH1; CHK1; CTT1; UNG1; MAG1; NTG2; GRX1; HOG1; SLN1	47	21
		**Gene Ontology Cellular Components**		
**nucleus [GO:0005634]**	0.0167	OGG1; XRS2; RAD4; MRE11; RAD9; CCS1; RAD51; HSP104; PHR1; MSH2; CHK1; UNG1; CDC28; TPK2; MAG1; NTG2; GRX1; HOG1	37	18
		**Gene Ontology Molecular Functions**		
**double-stranded DNA binding [GO:0003690]**	0.0137	XRS2; MRE11; RAD9; MSH2	4	4
**structure-specific DNA binding [GO:0043566]**	0.0195	XRS2; MRE11; RAD9; RAD51; MSH2	6	5
**kinase activity [GO:0016301]**	0.0465	MRE11; CHK1; CDC28; TPK2; HOG1; SLN1	9	6

Note: a: observed markers indicate proteins up-regulated with $PELI_{ORF}$ > 1.5.
